# Revealing Potential Diagnostic Gene Biomarkers Associated with Immune Infiltration in Patients with Renal Fibrosis Based on Machine Learning Analysis

**DOI:** 10.1155/2022/3027200

**Published:** 2022-04-20

**Authors:** Yu-Chao Sun, Zhen-Zhen Qiu, Fu-Li Wen, Jin-Quan Yin, Hao Zhou

**Affiliations:** ^1^Department of Clinical Medicine Research Center, The Fourth Affiliated Hospital, International Institutes of Medicine, Zhejiang University School of Medicine, Yiwu 322000, China; ^2^Department of Physical Education, Minjiang University, Fuzhou 350108, China; ^3^Laboratory of Basic Medicine, 900 Hospital of the Joint Logistics Team, PLA, Fuzhou 350025, China; ^4^Research Center, Bell Biotechnology Co, Changsha, Hunan, China; ^5^Department of Urology, The Fourth Affiliated Hospital, International Institutes of Medicine, Zhejiang University School of Medicine, Yiwu 322000, China

## Abstract

Chronic kidney disease is characterized by the development of renal fibrosis. The basic mechanisms of renal fibrosis have not yet been fully investigated despite significant progress in understanding the etiology of the disease. In this work, the researchers sought to identify potential diagnostic indicators for renal fibrosis. From the GEO database, we were able to acquire two gene expression profiles with publically available data (GSE22459 and GSE76882, respectively) from human renal fibrosis and control samples. 215 renal fibrosis specimens and 124 normal specimens were examined for differentially expressed genes (DEGs). The SVM-RFE and LASSO regression models were used to discover potential markers. CIBERSORT was applied to estimate the combined cohorts' immune cell fraction compositional trends in renal fibrosis. RT-PCR was used to examine the expression of ISG20 in renal fibrosis and healthy samples. In vitro experiments were applied to examine the function of ISG20 knockdown on the progression of renal fibrosis. In this study, we identified 24 DEGs. The result of LASSO and SVM-RFE identified nine critical genes. ROC assays confirmed the diagnostic value of the above nine genes for renal fibrosis. Immune cell infiltration analysis revealed that ISG20 and SERPINA3 were both found to be correlated with T cell follicular helper, neutrophils, T cell CD4 memory activated, eosinophils, T cell CD8, dendritic cell activated, B cell memory, monocytes, macrophage M2, plasma cells, T cell CD4 naïve, mast cell resting, B cell naïve, T cell regulatory, and NK cell activated. Finally, we observed that the expression of ISG20 and SERPINA3 was distinctly increased in renal fibrosis samples compared with normal samples. ISG20 siRNA significantly suppressed the progression of renal fibrosis in vitro. Overall, this study identified nine diagnostic biomarkers for renal fibrosis. ISG20 may be a novel therapeutic target of renal fibrosis.

## 1. Introduction

The importance of fibrotic diseases rises in a global awareness, as approximately 45% of all deaths in the Western world are related to various forms of fibrosis [1]. Chronic kidney disease (CKD) is a major public health issue that increases morbidity and death, and renal fibrosis is the most common endpoint and core pathological process of CKD [2, 3]. While renal fibrosis is thought to be a result of the epithelial-to-mesenchymal transition (EMT), this transition is not well understood [4, 5]. Renal biopsy is the only way to definitively diagnose kidney fibrosis [6, 7]. Patients with end-stage renal illness are more at risk because the procedure is invasive and can lead to bleeding problems [8]. Therefore, the quest for biomarkers of renal fibrosis that are more readily available and specific is important.

In recent years, high throughput sequencing, together with integrated bioinformatics assays, has been carried out for the identification of novel genes related to many types of diseases that might serve as diagnostic and prognostic markers [9, 10]. Since TXNDC5 is a key player in the development of cardiac fibrosis and has been shown to be highly expressed in kidney fibroblasts, it is reasonable to assume that targeting TXNDC5 in the treatment of kidney fibrosis and chronic kidney disease (CKD) could have therapeutic benefits [11]. Excessive production of BMP-7 in diabetic nephropathy-induced renal fibrosis could be prevented by downregulating miR-21, according to a study published in the Journal of Nephrology [12]. Renal fibrosis, for example, has been demonstrated to be linked to an increase in immune cell infiltration. Renal fibrosis-induced immune cell modulation has recently been shown to have an impact on renal function, offering new insights into the importance of immune regulation in chronic kidney disease [13, 14]. However, there have been few studies to date that have used CIBERSORT to study renal fibrosis and identify possible diagnostic indicators.

We retrieved two renal fibrosis microarray datasets from the GEO database for this study. A meta-data cohort was created by combining the two datasets via the combat function of the SVA package. The renal fibrosis and normal tissues were compared using a differentially expressed genes (DEGs) analysis. Renal fibrosis diagnostic biomarkers were filtered and identified using machine-learning techniques. Immune cells were quantified for the first time in renal fibrosis and healthy tissues using CIBERSORT, which was developed to analyze gene expression patterns [15]. To establish a foundation for future study, we investigated the link between biomarkers and invading immune cells.

## 2. Materials and Methods

### 2.1. Cell Culture

Human renal tubular epithelial cell lines (HK-2) were purchased from Procell Life Science&Technology (Hongshan, Wuhan, China) and maintained in RPMI-1640 medium (E600028, Sangon Biotech, Songjiang, Shanghai, China), supplemented with 10% FBS (Gibco, Guangzhou, Guangdong, China), 1% penicillin (Sangon Biotech, Songjiang, Shanghai, China), and 1% streptomycin in a humidified incubator with 5% CO_2_ at 37°C. TGF-1 (Procell Life Science&Technology, Hongshan, Wuhan, China) was used to produce a cellular model of renal fibrosis in HK-2, which were treated for 48 hours.

### 2.2. Cell Transfection

Lipofectamine® 2000 Transfection reagent was used to transfect HK-2 cells with either an ISG20 small interfering (si) RNA or a si-negative control (si-NC) (Sangon Biotech, Songjiang, Shanghai, China). At 6 × 10^6^/well cells per well, HK-2 cells were seeded into 6-well plates at 37°C for 24 hrs. Once the plate was filled, 1.5 ml media without serum or antibiotics was added to each well of the plate, followed by a mixture of 100 pmol ISG20 siRNA and Lipofectamine® 2000, which was incubated for 4-6 hours at 37°C with 5% CO_2_. qRT-PCR confirmed the effectiveness of the knockdown of genes.

### 2.3. RNA Extraction and Quantitative RT-PCR (qPCR)

In accordance with the manufacturer's recommendations, RNA was extracted and purified from cell lines using an RNAqueous Total RNA Isolation Kit (Sangon Biotech, Songjiang, Shanghai, China) and then sequenced. Reverse transcription was carried out with the help of a High-Capacity cDNA Reverse Transcription Kit (Applied Biosystems, USA) in order to quantify ISG20 expressions. QPCR was performed on an ABI Prism 7200 HT sequence detector (Applied Biosystems, China), along with a SYBR Green Master Mix kit (Solarbio, Tongzhou, Beijing, China). The expression levels of ISG20 were determined using the 2-Ct technique, which yields relative fold changes. GeneCopoeia provided the primers that were used in this study. The normalizer employed in this study was GAPDH. The sequences were as follows: ISG20 primers: 5′-CTCGTTGCAGCCTCGTGAA-3′ (forward) and 5′-CGGGTTCTGTAATCGGTGATCTC-3′ (reverse); SERPINA3 primers: 5′-CCTGAAGGCCCCTGATAAGAA-3′ (forward) and 5′-GCTGGACTGATTGAGGGTGC-3′ (reverse); GAPDH primers: 5′-GGAGCGAGATCCCTCCAAAAT-3′ (forward) and 5′-GGCTGTTGTCATACTTCTCATGG-3′ (reverse).

### 2.4. Western Blot

SDS-PAGE was used to separate the proteins from the lysate, and the lysate was then transferred to nitrocellulose membranes for additional investigation. Following that, a Western blot assay was performed in accordance with the established Western blot procedure. Furthermore, *β*-actin was employed as a cytosol protein marker, and Santa Cruz Biotechnology supplied the ISG20 antibody.

### 2.5. Data Acquisition

The data of renal fibrosis and healthy specimens were collected from the GEO datasets. There were 40 renal fibrosis and 25 normal samples in the GSE22459 datasets [16]. There are 175 renal fibrosis and 99 normal samples in GSE76882 datasets [17]. The DEGs were identified by the use of the above GEO datasets.

### 2.6. DEG Screening, Data Processing, and DEG Analysis

In order to detach batch effects from the two datasets, the combat functions of the SVA package were applied in conjunction with the merge function to create a metadata cohort. Background associations and differential expression assays between 215 renal fibrosis and 124 control samples were all performed using the limma package of R (http://www.bioconductor.org/limma/) as described above. Those samples having an adjusted false discovery rate (AFR) of less than 0.05 and a |log fold change (FC)| greater than 1.2 were determined to be DEG threshold points.

### 2.7. Functional Enrichment Analysis

For patients in the low- and high-risk categories, the “clusterProfiler” R package was used to conduct an enrichment analysis of pathways using the Gene Ontology (GO) and the Kyoto Encyclopedia of Genes and Genomes (KEGG). *P* values less than 0.05 were considered statistically significant for GO keywords and KEGG pathways. Using R's “clusterProfiler” and “DOSE” packages, we ran enrichment analysis for disease ontology (DO) terms on DEGs [18]. When comparing the renal fibrosis and control groups, we employed a technique called gene set enrichment analysis (GSEA). If a *P* 0.05 and a false discovery rate 0.05 were met, a gene collection was considered to be highly enriched.

### 2.8. Candidate Diagnostic Biomarker Screening

Two machine-learning methods were utilized to discover relevant prognostic indicators in order to predict the disease status. This is a regression analysis algorithm called the least absolute shrinkage and selection operator (LASSO) [19]. It uses regularization to improve the accuracy of the predictions. The “glmnet” package in R was used to run the LASSO regression algorithm and find the genes that were most important at separating renal fibrosis from normal samples. In machine learning, the support vector machine (SVM), a supervised machine-learning algorithm, was applied for regression and classification [20]. Because overfitting can happen when too many genes are chosen, an algorithm called RFE was used to choose the best genes from the metadata group. This helped to avoid overfitting. Thus, this necessitated the application of SVM-RFE in order to choose acceptable characteristics for the goal of finding a subset of genes with the greatest discriminative potential.

### 2.9. Assays of Immune Cellular Patterns in Microenvironment

The immune cell fractions of GEO samples were analyzed by the use of CIBERSORT [21]. CIBERSORT can quantify infiltrating immune cell fractions based on normalized patterns of gene expressions, which can be obtained from a variety of sources. It was submitted to the CIBERSOFT website where the standardized gene expression data collection could be found (https://cibersort.stanford.edu/index.php). CIBERSORT *P* values were calculated for each sample using Monte Carlo sampling to increase the accuracy of the algorithm. Only samples with CIBERSORT *P* values less than 0.05 were considered eligible for analysis.

### 2.10. Detection and Analysis of Gene-Immune Cell Correlations

Spearman's rank correlation assays were applied to investigate the associations between the identified gene markers and amounts of invading immune cells. Using the “ggplot2” program, we were able to display the results in the form of a chart [22].

### 2.11. Statistical Analysis

All statistical analyses were operated via the R software (version 3.6.2) and GraphPad Prism 8. LASSO regression assays were conducted by the use of the “glmnet” package, and the SVM algorithm was carried out by the use of e1071 package. The diagnostic efficacies of the markers were examined by the use of ROC curves. It was feasible to establish a link between the expressions of genes and the infiltration of immune cells with the help of Spearman's correlation. The Wilcox test was applied for the determination of whether there were any differences between the two groups. A statistically significant difference was defined as a*P*value less than 0.05.

## 3. Results

### 3.1. Identification of DEGs in Renal Fibrosis

**Figure 1:**
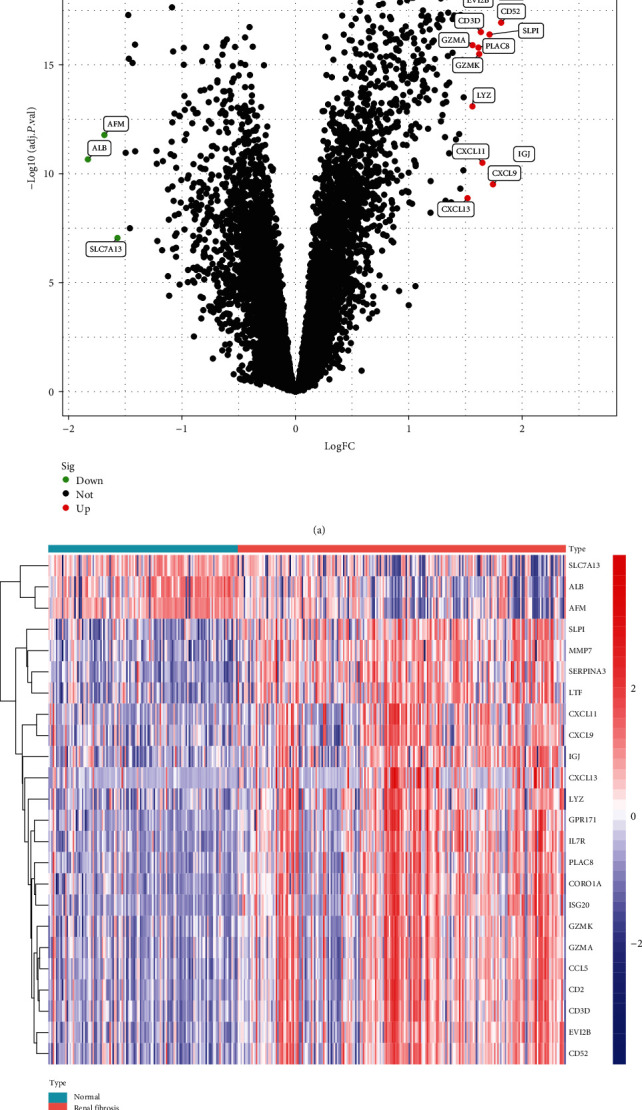
Volcano plot (a) and heat map (b) of differentially expressed genes between renal fibrosis specimens and healthy specimens.

Data from 215 renal fibrosis samples, as well as 124 normal specimens from two GEO (GSE22459 and GSE76882) were used in our investigation, which was conducted in a retrospective manner. We performed a limma package analysis after removing any batch effects from the metadata and found that the DEGs were significantly higher than the baseline. There were a total of 24 DEGs. There was a significant increase in 21 genes and a significant decrease in 3 genes (Figures 1(a) and 1(b)).

### 3.2. Functional Correlation Analysis

**Figure 2:**
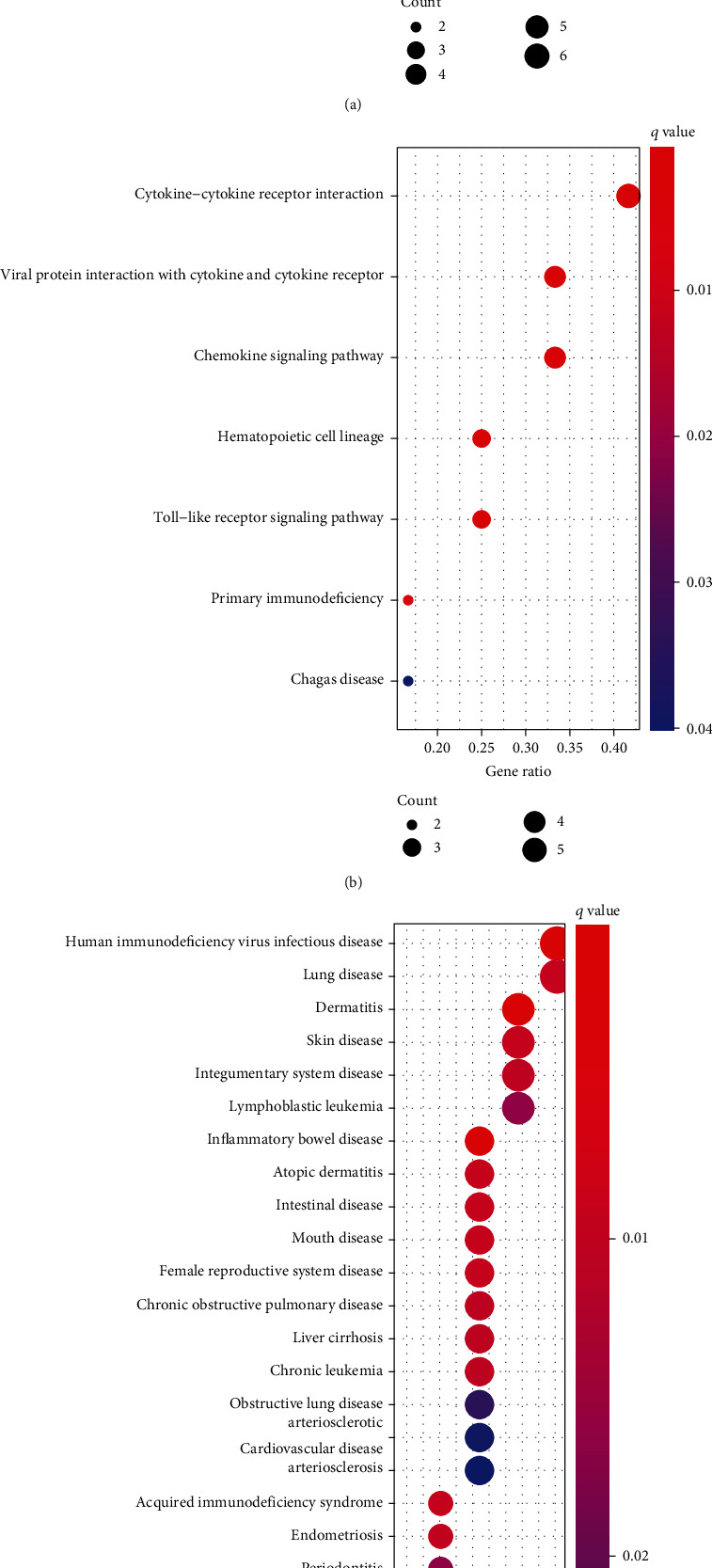
GO, KEGG, and DO pathway enrichments of differentially expressed genes: (a) GO enrichment; (b) KEGG pathway enrichment; (c) DO enrichment analysis.

**Figure 3:**
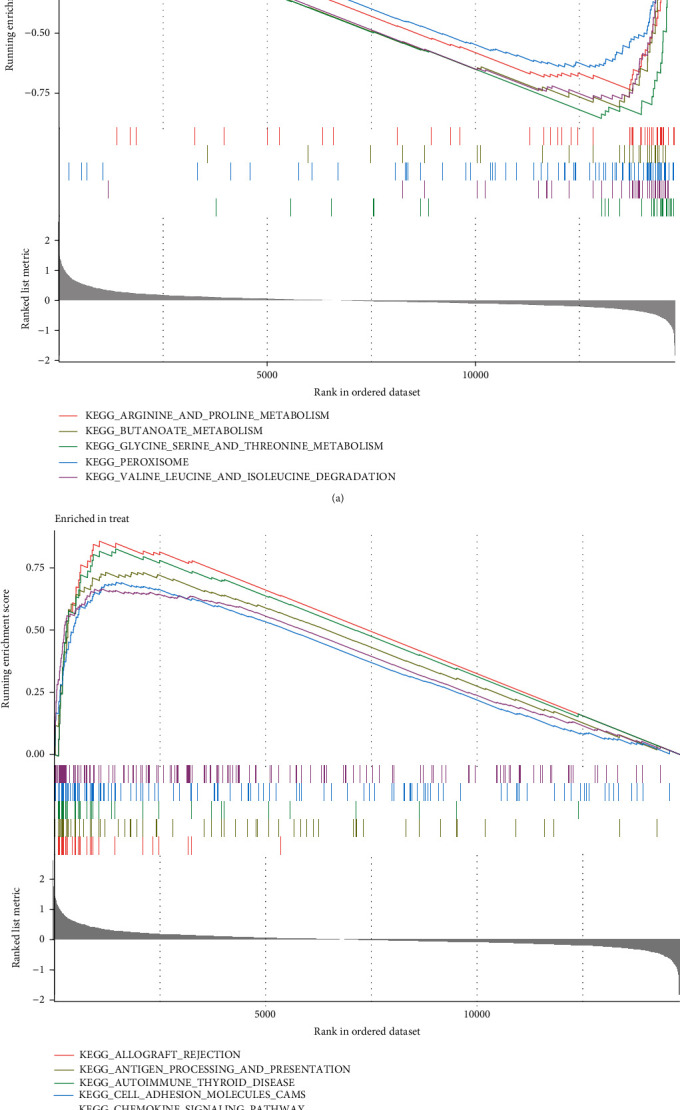
(a, b) Enrichment analyses via gene set enrichment analysis.

To divide into the potential functions of the above DEGs in human, we performed GO and KEGG assays. The findings of GO assays indicated that the DEGs were mainly enriched in antimicrobial humoral response, response to lipopolysaccharide, secretory granule lumen, cytoplasmic vesicle lumen, chemokine activity, and chemokine receptor binding (Figure 2(a)). The results of KEGG revealed that the DEGs were mainly involved in cytokine-cytokine receptor interaction, chemokine signaling pathway, hematopoietic cell lineage, Toll-like receptor signaling pathway, primary immunodeficiency, and Chagas disease (Figure 2(b)). DO pathway enrichment assays revealed that diseases enriched by DEGs were mainly related to human immunodeficiency virus infectious disease, dermatitis, lymphoblastic leukemia, inflammatory bowel disease, and chronic leukemia (Figure 2(c)). The GSEA indicated that the enriched pathways largely involved ANTIGEN_PROCESSING_AND_PRESENTATION, AUTOIMMUNE_THYROID_DISEASE, ALLOGRAFT_REJECTION, CELL_ADHESION_MOLECULES_CAMS, and CHEMOKINE_SIGNALING_PATHWA (Figures 3(a) and 3(b)).

### 3.3. Identification and Validation of Diagnostic Markers

**Figure 4:**
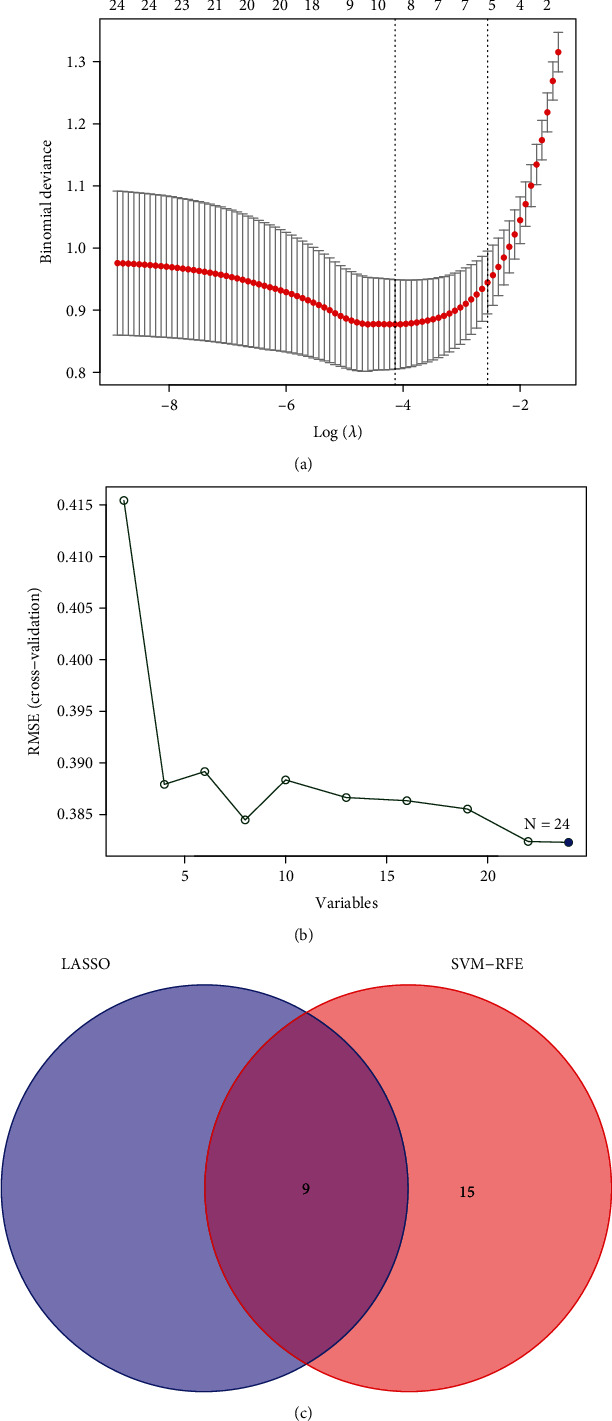
Selection of potential renal fibrosis diagnostic biomarkers. (a) Tuning feature selection in the LASSO. (b) The SVM-RFE technique was used to pick biomarkers. (c) In this Venn diagram, the SVM-RFE and LASSO algorithms are shown to have four diagnostic markers between them.

**Figure 5:**
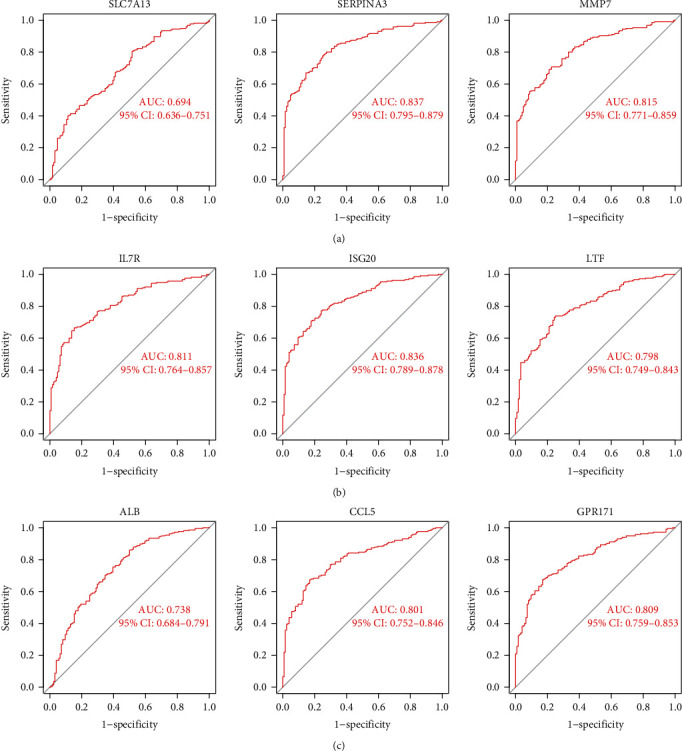
ROC assays were applied to determine the diagnostic value of the critical genes, including (a) SLC7A1, SERPINA, and MMP7; (b) IL7Rs, ISG20, and LTF; and (c) ALB, CCL5, and GPR171.

To find potential biomarkers, researchers utilized two separate algorithms. By utilizing the LASSO regression algorithm, the DEGs were reduced to nine variables, which were identified as diagnostic biomarkers of renal fibrosis (Figure 4(a)). The SVM-RFE approach was used to narrow down the DEGs to a subset of 24 genes (Figure 4(b)). The nine overlapping features (ALB, CCL5, GPR171, IL7R, ISG20, LTF, MMP7, SERPINA, and SLC7A1) between these two algorithms were eventually picked (Figure 4(c)). Then, we performed ROC assays to explore the diagnostic value of the above nine genes for renal fibrosis. As shown in Figures 5(a)–5(c), we observed that all nine genes are powerful discrimination ability for renal fibrosis.

### 3.4. Correlation of ISG20 and SERPINA with the Proportion of Immune Cells

**Figure 6:**
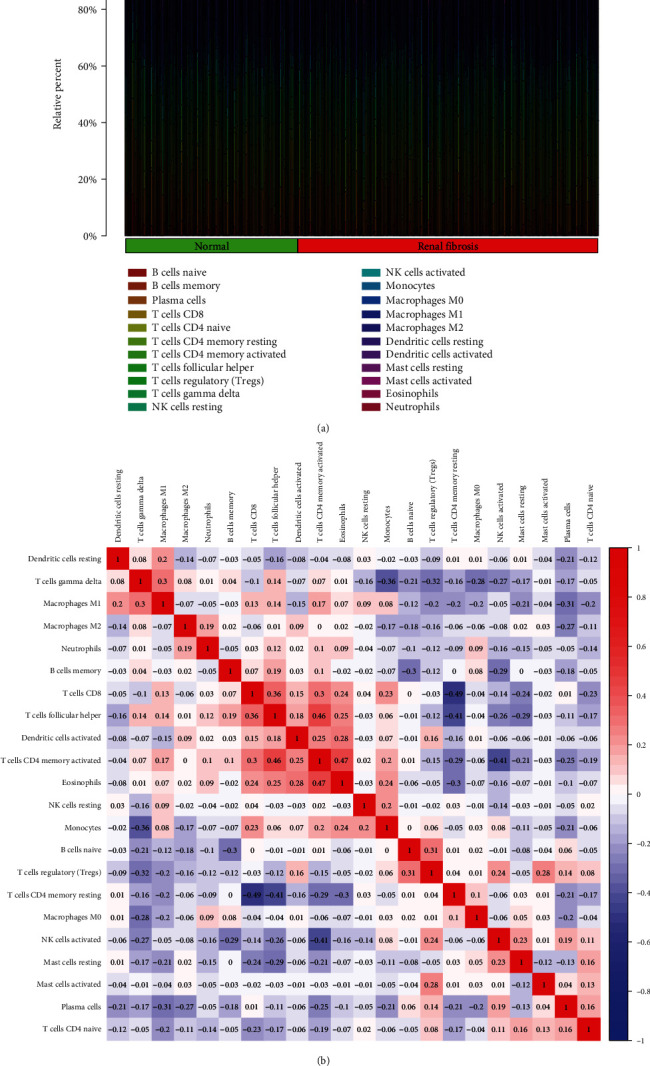
According to CIBERSORT, the proportion of infiltrating immune cells. (a) The total number of immune cells found in each sample is reported. (b) A heat map was constructed to illustrate the link between the 21 different types of immune infiltrate cells that were identified.

**Figure 7:**
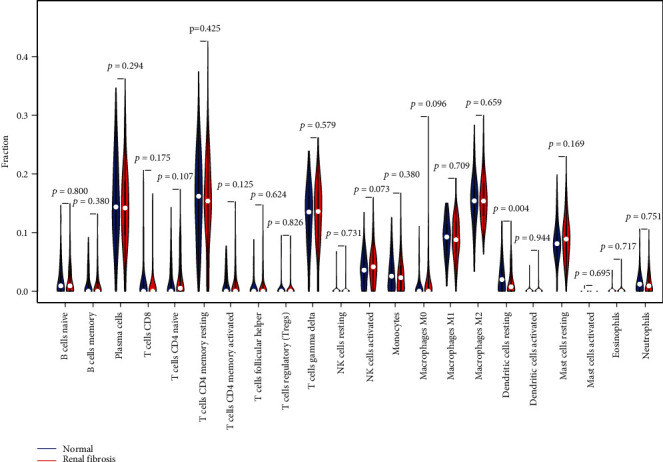
Between renal fibrosis and normal kidney tissues, 22 immune cell subtypes were analyzed and compared.

**Figure 8:**
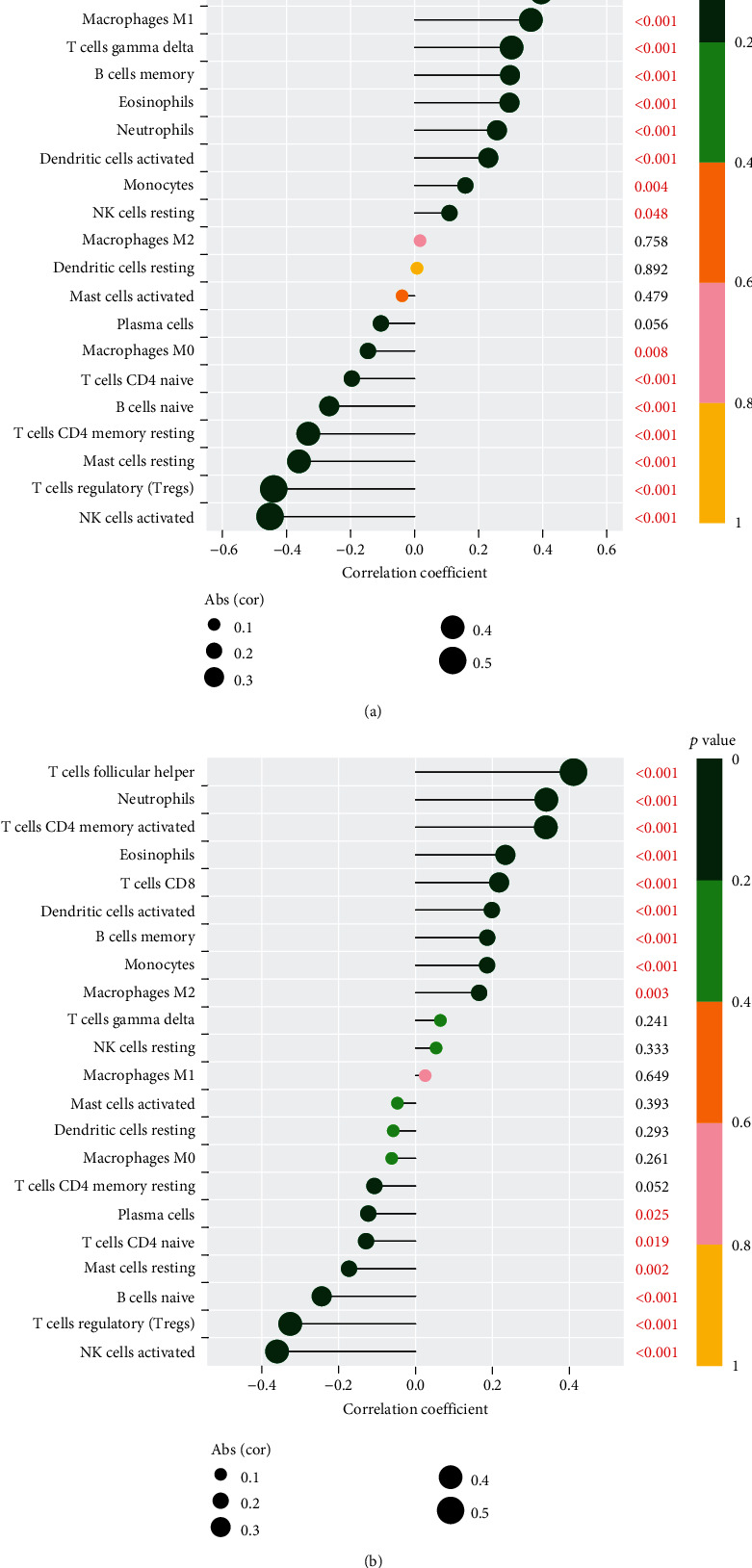
Correlation between (a) ISG20, (b) SERPINA3, and infiltrating immune cells in renal fibrosis.

For the purpose of determining the proportion of tumor-infiltrating immune subsets, the CIBERSORT method was used in conjunction with 21 different immune cell profiles built from samples of renal fibrosis in order to determine the relationship between ISG20 and SERPINA expression and the immune microenvironment (Figures 6(a) and 6(b)). The expressing pattern of immune cells is shown in Figure 7(a). By performing correlation analysis between ISG20, SERPINA3, and immune cells, ISG20 and SERPINA3 were all found to be correlated with T cell follicular helper, neutrophils, T cell CD4 memory activated, eosinophils, T cell CD8, dendritic cell activated, B cell memory, monocytes, macrophage M2, plasma cells, T cell CD4 naïve, mast cell resting, B cell naïve, T cell regulatory, and NK cell activated (Figures 8(a) and 8(b)).

### 3.5. ISG20 siRNA Significantly Suppressed the Progression of Renal Fibrosis In Vitro

**Figure 9:**
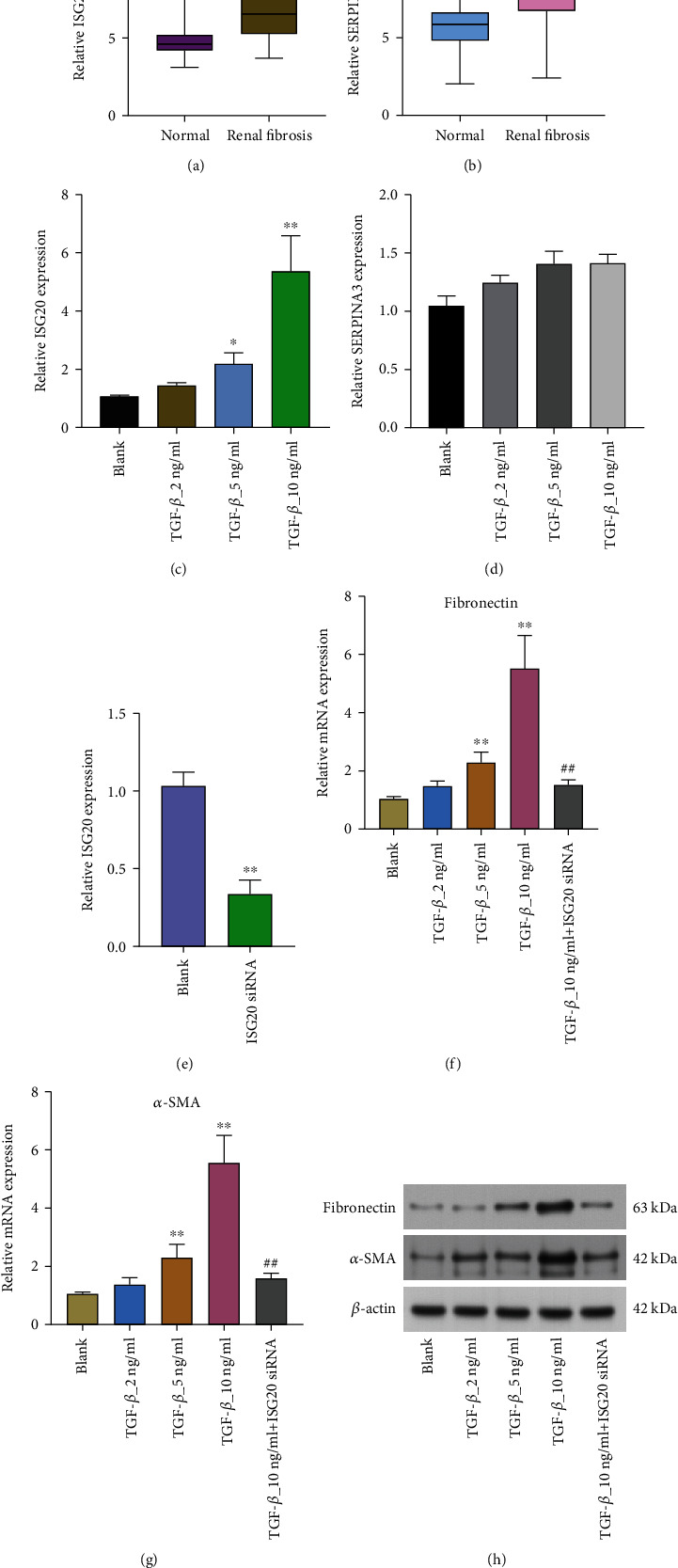
In vitro, suppression of the ISG20 gene significantly reduced the evolution of renal fibrosis. (a, b) In patients with renal fibrosis, the expression of ISG20 and SERPINA3 was found to be upregulated in a unique manner. (c, d) For 48 hours, HK-2 cells were exposed to concentrations of 2, 5, or 10 ng/ml TGF-1. qRT-PCR was used to detect the expressions of ISG20 and SERPINA3 in HK-2 cells. (e) ISG20 siRNA was transfected into HK-2 cells. Subsequently, using RT-PCR, we discovered that ISG20 was expressed in HK-2 cells. (f, g) RT-PCR was used to find out how much mRNA fibronectin and *α*-SMA were making in HK-2 cells. (h) The protein expressions of fibronectin and *α*-SMA in HK-2 cells were determined by western blot.

The expression of ISG20 and SERPINA3 was distinctly increased in renal fibrosis samples compared with normal samples (Figures 9(a) and 9(b)). In order to simulate renal fibrosis in vitro, HK-2 cells were treated with different doses of TGF- (2, 5, or 10 ng/mL) for varied periods of time. As displayed in Figure 9(c), 5 or 10 ng/mL TGF-*β*1 notably promoted the expressions of ISG20 in HK-2. However, the expression of SERPINA3 remained unchanged (Figure 9(d)). Thus, we focused on ISG20. Aside from that, the levels of ISG20 in HK-2 cells were dramatically reduced by the ISG20 siRNA treatment (Figure 9(e)). Moreover, there was a dosage response in HK-2 when TGF-1 increased the expressions of fibrotic proteins (*α*-SMA and fibronectin). Importantly, the increase of fibronectin and *α*-SMA caused by TGF-1 was greatly inhibited by ISG20 siRNA, which was also effective (Figures 9(f)–9(h)).

## 4. Discussion

When the kidneys are damaged, the fibrosis process begins, and the disease continues to progress [23, 24]. Fibrosis may play a causal role in the course of renal disease, but this is still up for debate, according to the research [25]. Biomarkers of fibrosis are essential to understanding the course of CKD because they can provide critical information in a noninvasive manner [26, 27]. Patients with an increased risk of developing chronic kidney disease (CKD) could benefit from clinical trials in which they would be selected from a trustworthy pool of fibrosis biomarkers [28, 29].

Using two GSE datasets, we found 24 genes that differed between renal fibrosis and healthy tissue samples in this study. To learn more about how DEGs work, researchers used enrichment studies of the DO pathway. The results showed that disorders enriched by DEGs were predominantly connected with HIV infection, lymphoblastic leukemia, dermatitis, inflammatory bowel disease, and AIDS. The GSEA results demonstrated that the enriched pathways mainly involved ALLOGRAFT_REJECTION, ANTIGEN_PROCESSING_AND_PRESENTATION, AUTOIMMUNE_THYROID_DISEASE, CELL_ADHESION_MOLECULES_CAMS, and CHEMOKINE_SIGNALING_PATHWA. In the case of renal fibrosis, these findings strongly suggest that the immune response is an important factor.

Two separate techniques were used to narrow down the list of potential biomarkers for renal fibrosis diagnosis. The nine overlapping features (ALB, CCL5, GPR171, IL7R, ISG20, LTF, MMP7, SERPINA, and SLC7A1) between these two algorithms were ultimately selected. The potential function of the above genes has been reported in renal fibrosis. For instance, the CCL5-CCR5 axis and the TGF-1/Smad/Snail signaling pathways were inhibited by inducing myeloid-derived suppressor cells in vitro and in vivo, and both methods reduced kidney fibrosis [30]. Smad4 deacetylation and inhibition of MMP7 expression by upregulated SIRT1 in RSV reduced kidney damage and fibrosis, which was related to the upregulation of SIRT1 by RSV [31]. However, the role of several genes in renal fibrosis remains a mystery. According to the results from ROC assays performed in this work, the above nine genes have the potential to be employed as new biomarkers for renal fibrosis.

CIBERSOTR was used to examine the types of immune cell infiltration in renal fibrosis and normal samples. Immune cell subtypes were discovered to play an essential role in renal fibrosis-related biological processes as a result [32, 33]. By performing correlation analysis between ISG20, SERPINA3, and immune cells, ISG20 and SERPINA3 were all found to be correlated with T cell follicular helper, neutrophils, T cell CD4 memory activated, eosinophils, T cell CD8, dendritic cell activated, B cell memory, monocytes, macrophage M2, plasma cells, T cell CD4 naïve, mast cell resting, B cell naïve, T cell regulatory, and NK cell activated. In fact, it has already been demonstrated that inflammatory and immune circulatory cells play a crucial role in the progression of renal fibrosis [34, 35]. As previously said, the substantial data, together with our current findings, has revealed that numerous types of invading immune cells play critical roles in the progression of renal fibrosis and should be the focus of future research.

Finally, we performed in vitro assays to study the function of ISG20 and SERPINA. We found that both ISG20 and SERPINA exhibited a higher level in renal fibrosis than normal specimens. However, 5 or 10 ng/mL TGF-*β*1 only promoted the expressions of ISG20 in HK-2, while the expression of SERPINA3 remained unchanged. Thus, we further focused on ISG20. The results of Western blot and PCR revealed that knockdown of ISG20 distinctly suppressed the expression of the expressions of fibrotic proteins (*α*-SMA and fibronectin), indicating ISG20 promoted the progression of renal fibrosis.

However, there are a few limitations in this study. There is still a need for prospective samples to be validated in our investigation, as all of the cases were retrospective. This study did not include any patients who had received immunotherapy; hence, the capacity of the genes to predict immunotherapy response was assessed indirectly. Prospective studies with sufficient power are still required.

## 5. Conclusion

In summary, ALB, CCL5, GPR171, IL7R, ISG20, LTF, MMP7, SERPINA, and SLC7A1 were identified as diagnostic biomarkers of renal fibrosis. ISG20 and SERPINA3 were associated the levels of most immune cells. Patients with renal fibrosis may benefit from our findings since they may provide a clinically helpful tool for better prognostic management as well as for optimizing the accompanying treatment.

## Data Availability

The data used to support the findings of this study are available from the corresponding author upon request.
